# Supplementary material for: Does Sex Matter in the Link Between Self-Efficacy and Mediterranean Diet Adherence in Adolescents? Insights from the EHDLA Study
Source: Nutrients. 2025 Feb 28;17(5):880. doi: 10.3390/nu17050880 (PMC11902149; doi:10.3390/nu17050880)
Supplement: Supplementary file 1 [file nutrients-17-00880-s001.zip › nutrients-3483240-supplementary.pdf]

Supplementary material

Table S1. Generalized linear model assessing the associations of self-efficacy and covariates with the Mediterranean Diet Quality Index score for children and adolescents for boys.

	Outcome: KIDMED (score)				
Predictor	*B*	SE	95% CI		*p* value
			Lower	Upper	
Self-efficacy (per point)	0.01	0.01	-0.01	0.02	0.419
Age (per year)	0.06	0.10	-0.13	0.25	0.527
FAS-III (score)	-0.03	0.07	-0.17	0.10	0.660
YAP-S physical activity (per point)	0.40	0.21	-0.01	0.81	0.058
YAP-S sedentary behaviors (per point)	-0.92	0.23	-1.38	-0.47	<0.001
Overall sleep duration (per hour)	0.15	0.17	-0.19	0.49	0.378
Body mass index (per kg/m^2)	0.02	0.03	-0.04	0.07	0.513
Energy intake (per 1000 kcal)	0.24	0.09	0.06	0.41	0.009

B, unstandardized beta coefficient; CI, confidence interval; FAS-III, Family Affluence Scale-III; KIDMED, KIDMED, Mediterranean Diet Quality Index for children and adolescents; SE, standard error; YAP-S, Spanish Youth Activity Profile.

Table S2. Generalized linear model assessing the associations of self-efficacy and covariates with the Mediterranean Diet Quality Index for children and adolescents for girls.

	Outcome: KIDMED (score)				
Predictor	*B*	SE	95% CI		*p* value
			Lower	Upper	
Self-efficacy (per point)	0.03	0.01	0.02	0.04	<0.001
Age (per year)	0.08	0.09	-0.10	0.26	0.371
FAS-III (score)	0.12	0.06	-0.01	0.25	0.067
YAP-S physical activity (per point)	0.72	0.21	0.31	1.12	0.001
YAP-S sedentary behaviors (per point)	-0.82	0.24	-1.28	-0.35	0.001
Overall sleep duration (per hour)	0.12	0.16	-0.19	0.42	0.459
Body mass index (per kg/m^2)	0.01	0.03	-0.05	0.07	0.710
Energy intake (per 1000 kcal)	0.07	0.06	-0.04	0.18	0.223

B, unstandardized beta coefficient; CI, confidence interval; FAS-III, Family Affluence Scale-III; KIDMED, KIDMED, Mediterranean Diet Quality Index for children and adolescents; SE, standard error; YAP-S, Spanish Youth Activity Profile.
